# Proliferative heterogeneity of murine epithelial cells in the adult mammary gland

**DOI:** 10.1038/s42003-018-0114-7

**Published:** 2018-08-13

**Authors:** Mona Shehata, Paul D. Waterhouse, Alison E. Casey, Hui Fang, Lee Hazelwood, Rama Khokha

**Affiliations:** 1 Princess Margaret Cancer Centre, University Health Network, Toronto, ON Canada M5G 1L7; 2 Cancer Research UK Cambridge Institute, University of Cambridge, Li Ka Shing Centre, Cambridge, CB2 0RE UK; 3 Present Address: Medical Research Council Cancer Unit, University of Cambridge, Hills Road, Cambridge, CB2 0XZ UK

## Abstract

Breast cancer is the most common cancer in females. The number of years menstruating and length of an individual menstrual cycle have been implicated in increased breast cancer risk. At present, the proliferative changes within an individual reproductive cycle or variations in the estrous cycle in the normal mammary gland are poorly understood. Here we use Fucci2 reporter mice to demonstrate actively proliferating mammary epithelial cells have shorter G1 lengths, whereas more differentiated/non-proliferating cells have extended G1 lengths. We find that cells enter into the cell cycle mainly during diestrus, yet the expansion is erratic and does not take place every reproductive cycle. Single cell expression analyses feature expected proliferation markers (*Birc5, Top2a*), while HR+ luminal cells exhibit fluctuations of key differentiation genes (*ER, Gata3*) during the cell cycle. We highlight the proliferative heterogeneity occurring within the normal mammary gland during a single-estrous cycle, indicating that the mammary gland undergoes continual dynamic proliferative changes.

## Abstract (web summary)

Mona Shehata et al. find that cell proliferation within the mouse mammary gland is highly heterogeneous, both between and within estrous cycles. Their study highlights the need for further understanding of the link between human menstrual cycle numbers and breast cancer risk.

## Introduction

The mammary gland is an epithelial tree of ducts and lobular structures, embedded in an adipose-rich stroma. Each duct and lobuloalveolar unit constitutes a bilayered outer basal cell layer and an inner hormone receptor-positive (HR+) and -negative (HR−) luminal cell layer. Mammary gland development is highly dependent on the circulating hormones oestrogen and progesterone, which induce mammary morphogenesis during distinct stages of the reproductive cycle^1^. These hormones act on HR+ luminal cells to elicit secretion of mitogenic paracrine signalling factors, which influence neighbouring HR− luminal and basal cells and induce rounds of proliferation. Cyclical surges in progesterone have also been shown to dramatically increase stem cell capacity and epithelial cell numbers in the adult breast^2–4^. Although hormonal signals can cause the majority of epithelial cells to proliferate, the number of proliferating cells varies greatly in the mammary gland between reproductive cycles^5,6^. At present, the proliferative changes within an individual reproductive cycle or variations in cell cycle length in the normal mammary gland are poorly understood. In this study, we utilised a combination of in vitro and in vivo functional assays and single cell genomics of the normal mammary epithelium to show the proliferative changes that occur during one estrous cycle. We demonstrate that most mammary epithelial cells proliferate during a specific phase of the estrous cycle. We also show that this expansion of the mammary gland does not occur during every estrous cycle and that there is substantial proliferative heterogeneity within the mammary gland. The proliferative activity during a cell cycle relies heavily on the length of time spent within the G1 phase showing the proliferative heterogeneity occurring in the mammary gland.

## Results

### Gene expression during the cell cycle phases

**Fig. 1:**
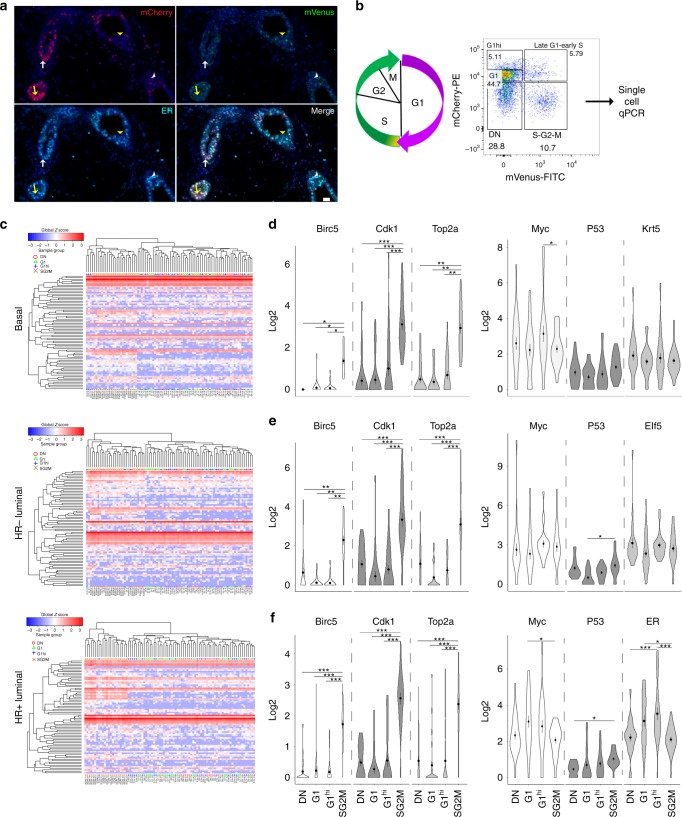
Single cell gene expression analysis of cycling mammary epithelial cells. **a** Immunofluorescence staining for mCherry to detect cells in G1 (red; arrows) and mVenus to detect S/G2/M (green; arrowheads) phases of the cell cycle in intact mammary glands. ERα (cyan) marker was used to discriminate between the HR− (yellow arrow/arrowheads) and HR+ (white arrow/arrowheads) luminal cells. Scale bar = 25 µm. **b** Schematic of gating strategy for single cell mammary epithelial cell analysis. **c** Heat maps showing unsupervised hierarchical clustering of gene expression of cells in G1, G1^hi^, S/G2/M and DN of the cell cycle, of 96 single cells from each of the basal, HR− and HR+ luminal compartments. Coloured symbols above heat map correspond to the cell cycle population. White to red intensity suggests middle to high expression, whereas white to blue suggests low to no expression. Violin plots show the distribution of cell cycle/mammary-related gene expression of single cells from the different cell cycle phases of the (**d**) basal, (**e**) HR− luminal and **f** HR+ luminal populations. Two-way ANOVA analysis with Tukey’s multiple comparison test was used to determine gene expression changes between different cell cycle phases. *, **, *** represent statistical significant at *P* < 0.05, *P* < 0.01, *P* < 0.001, respectively. No asterisks, statistically not significant

Specific mammary-related genes determine the lineage, however it is not known whether these mammary-specific lineage-determinants change during a cell cycle to determine cell fate. Thus we hypothesise that as cells enter into the cell cycle, changes in expression of key mammary-related genes will dictate progenitor activity. In order to accurately and precisely determine when mammary epithelial cells enter into a proliferative state, we needed a system to determine the cell cycle phases, in vivo, in real time. We employed the Fucci2 reporter mouse to visualize mammary epithelial cells in specific phases of the cell cycle. In the Fucci2 system, mCherry-hCdt1 (30/120) is expressed during G1, while mVenus-hGem (1/110) is expressed during S/G2/M phase of the cell cycle^7^. Additionally, both colours disappear during cytokinesis and very early in G1^7^. We first confirmed the presence of mCherry in G1 or mVenus in S/G2/M cells (Fig. 1a; Supplementary Fig. 1a) in intact mammary glands by immunofluorescent staining. To investigate the dynamic changes that occur in mammary cells throughout the cell cycle, we employed a microfluidics approach (Fluidigm) to perform multiplex gene expression analysis of freshly purified, flow sorted basal, HR− luminal and HR+ luminal cells. We performed single cell qPCR from these three mammary cell populations, each taken at G1, G1 high (G1^hi^) and S/G2/M phases of the cell cycle (Fig. 1b). We observed a novel population of cells, denoted double negative (DN), which have negative/lower levels of mCherry and are negative for mVenus expression (Fig. 1b). We queried genes altered in S/G2/M or across individual mammary cell lineages, as well as mammary cell fate determinants.

Unsupervised hierarchical clustering and principle component analysis (PCA) showed that expression of our target gene sets could distinguish S/G2/M mVenus-positive cells from cells in other cell cycle phases (Fig. 1c, Supplementary Fig. 1b). The heat maps and PCA plots of the DN population closely resemble G1/G1^hi^ cells, strongly suggesting that DN contains newly generated daughter cells. Cell cycle-specific genes (*Bric5*, *Cdk1*, *Top2a*) were all consistently upregulated during S/G2/M in all mammary epithelial populations (basal, HR− luminal, HR+ luminal). Interestingly, mammary-related genes did not noticeably change at the transcription level throughout the cell cycle in HR− luminal and basal cells; these included genes involved in cell proliferation (*Myc*, *Trp53*, *Ccnd1*), cell fate decision (*Notch2*, *Wnt4*, *FoxA1*) and genes encoding known mammary subset markers (*Id4*, *Krt5*, *Pten*, *Aldh1a3*, *Elf5*; Fig. 1d–f, Supplementary Fig. 2), suggesting that active proliferation/progenitor effects require constant expression of these genes throughout the cell cycle in order for cells not to be directed towards differentiation. Alternatively, entry into the cell cycle may not be entirely dependent on expression changes of tested mammary-related genes. Notably, HR+ luminal cells showed a different trend. We noted elevated levels of Gata3, ER, PR, FoxA1 and Cyclin D1 during G1/G1^hi^ phases in this population indicating a more differentiated status (Fig. 1f, Supplementary Fig. 2b,d), consistent with the reported increased Gata3 and FoxA1 expression in differentiated HR+ luminal cells^8,9^. These data confirmed the utility of the Fucci2 model to study mammary gland cell cycle dynamics.

### Actively proliferating cells have shorter G1 lengths

**Fig. 2:**
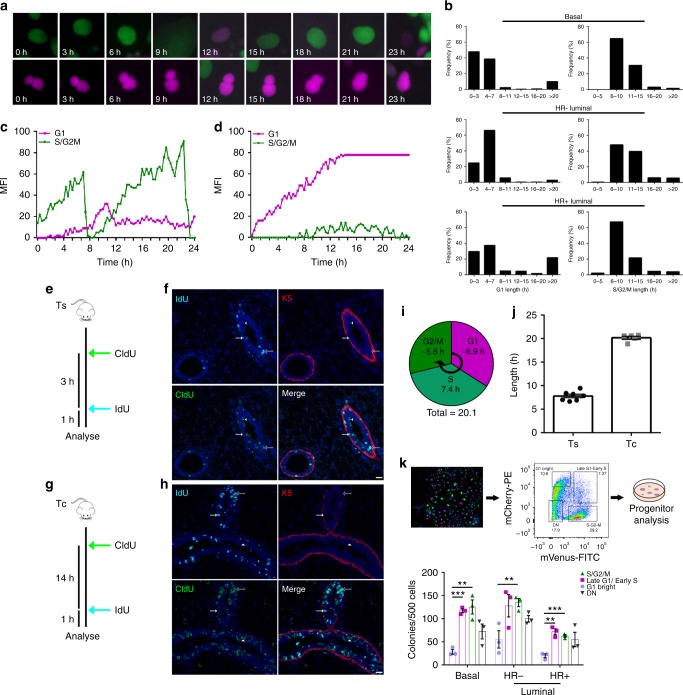
Diversity of cell cycle lengths in mammary epithelial cells. **a** Representative time-lapse images of the cell cycle determined by tracking Fucci2 fluorescence over 24 h from a cycling cell (upper panels) and a non-cycling cell residing in G1 (lower panels). **b** Histogram showing the distribution of G1 length (*n* = 250–300 segments) and S/G2/M length (*n* = 130–200 segments) in the basal, HR− and HR+ luminal cells (130–300 cells measured from three independent experiments). Changes in G1 (magenta) or S/G2/M (green) fluorescence intensity during 24 h imaging in a representative (**c**) proliferative or (**d**) differentiated cell, *n* = 130–300 cells measured over three independent experiments. **e** Schematic of Ts workflow. **f** Representative immunofluorescent images of CldU+ (green) IdU+ (cyan) cells, with basal marker Keratin 5 (K5, red). Scale bar = 25 µm. **g** Schematic of Tc workflow. **h** Representative immunofluorescent images of CldU+ (green) IdU+ (cyan) cells, with basal marker K5 (red). Arrows show IdU+, arrowheads show CldU+ and hollow arrows show CldU+IdU+ cells. Scale bar = 25 µm. **i** The diagram depicts the calculated/estimated length of the different cell cycle phases as a mean value of all proliferating cells in the mammary epithelium in vivo. **j** Bar graph showing the length of Ts (*n* = 7) and Tc (*n* = 5); mean ± SEM. **k** Schematic of the CFC assay and bar graph depicting clonogenic capacity at different phases of the cell cycle for basal, HR− and HR+ luminal colonies

It has been shown that the duration of particular cell cycle stages plays a major role in determining the cell fate choice. Specifically, the length of G1 has been implicated in controlling differentiation in neurons^10^ and embryogenesis^11,12^. We measured the length of time mammary epithelial cells spend in G1/G1^hi^ and S/G2/M by time-lapse microscopy on flow purified basal, HR− and HR+ luminal cells cultured in colony forming capacity (CFC) assays. On day 4 of the CFC assay, between 130 and 300 cells were tracked over a period of 24 h to record a full cell division, with all Fucci2 mammary epithelial populations displaying the expected transition from mCherry (magenta) to mVenus (green) fluorescence upon cell cycle progression (Fig. 2a, Supplementary Fig. 3a-b). In all populations, the majority of cells spent an average of 5 h in G1 and 10 h in S/G2/M. However a small proportion of cells resided in G1 for extended periods of time that ranged >20 h. We observed that 20% of HR+ luminal and 10% of basal cells had extended G1 lengths, suggesting a more differentiated phenotype exists within these cell populations (Fig. 2b). Interestingly, cells that resided in G1 for a shorter period of time had lower mCherry intensities and were frequently observed to progress into the next DNA replication phase (Fig. 2c, Supplementary Fig. 3c-e). Cells that had a longer G1 cycle had greater mCherry intensity and did not enter the next cycling phase within the 24-h window (Fig. 2d, Supplementary Fig. 3f-h).

We next wanted to determine the length of the cell cycle in vivo by administering CldU (5-chloro-2’-deoxyuridine) and IdU (5-Iodo-2’-deoxyuridine) (Fig. 2e–h). Both dyes incorporate into DNA during replication and the ratio of double-positive/single-positive cell numbers enumerates S phase (Ts) or total cell cycle (Tc) length, providing a basis for projecting the lengths of G1 and G2/M (Fig. 2i). We found that mammary epithelial cells spent an average of 7.4 h in S phase and generally took 20 h to complete each cell division. Overall, we noted cell cycle time durations in vivo were slightly longer than in vitro mammary cell analyses. The G1 length in vivo was ~7 h, and in vitro G1 lengths were on average 5 h. When comparing in vivo to in vitro S/G2/M lengths were 13, and 10 h, respectively. We observe similar G1:S/G2/M ratios between in vivo and in vitro indicating that in vitro assessment in CFCs reflects endogenous cell cycle dynamics (Fig. 2i–j).

**Fig. 3:**
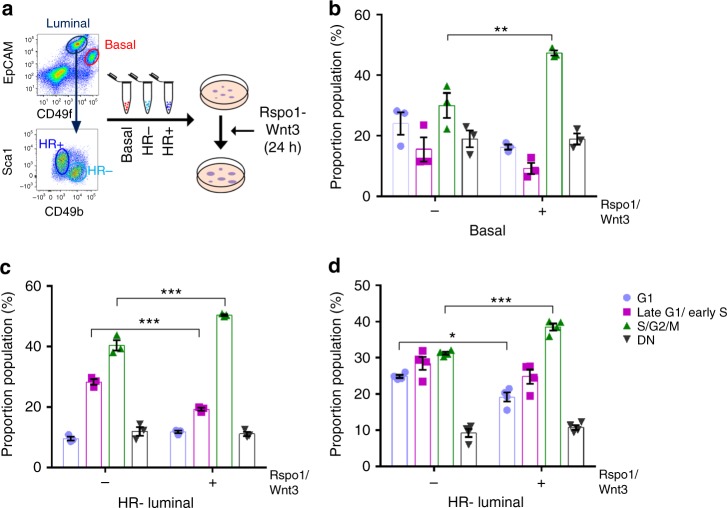
R-Spondin1-Wnt3 drives cells into a proliferative state. **a** Schematic of Rspo1-Wnt3 administration to mammary cells. Bar graphs indicate the proportion of cells in the different phases of the cell cycle within the (**b**) basal, (**c**) HR−, and **d** HR+ luminal colonies after 24 h treatment with DMSO or Rspo1-Wnt3 (mean ± SEM; *n* = 3). Two-way ANOVA analysis with Tukey’s multiple comparison test was used to determine changes in cell cycle proportions between DMSO and Rspo1-Wnt3 treatments within the different mammary epithelial cell types and changes of progenitor capacity between different cell cycle phases. *, **, *** represent statistical significant at *P* < 0.05, *P* < 0.01, and *P* < 0.001, respectively

To test whether mammary epithelial cells with a longer G1 phase were more differentiated, we sorted mCherry bright (G1 bright), double-positive (late G1/early S), mVenus (S/G2/M phase) and the DN populations from basal, HR− or HR+ luminal cell colonies and assayed them for their progenitor potential. The mCherry bright cells had overall lower progenitor potential compared to late G1/early S, S/G2/M, or DN subsets (Fig. 2k). Joshi et al. demonstrated that exposure to R-Spondin1 (Rspo1) resulted in increased proliferation^13^, while another study showed that addition of Wnt3 resulted in increased colony numbers^14^. Thus, we reasoned that addition of these two compounds would stimulate mammary epithelial cells to proliferate in culture. As differentiated cells appear to have longer G1 lengths, we reasoned that forcing a proliferative phenotype could increase the number of cells in S/G2/M phases. Adding Rspo1 and Wnt3 for 24 h appeared to decrease the number of HR+ cells in G1 and increase the number of cells in S/G2/M, in all populations (Fig. 3). Thus, the mammary gland may use the length of G1 as the commitment stage to enter into the cell cycle, where a shorter G1 enables cells to progress towards a proliferative state more readily and a longer G1 allows accumulation of differentiation factors that cause a quiescent/differentiated state.

### Two diestrus states exist within the mammary gland

**Fig. 4:**
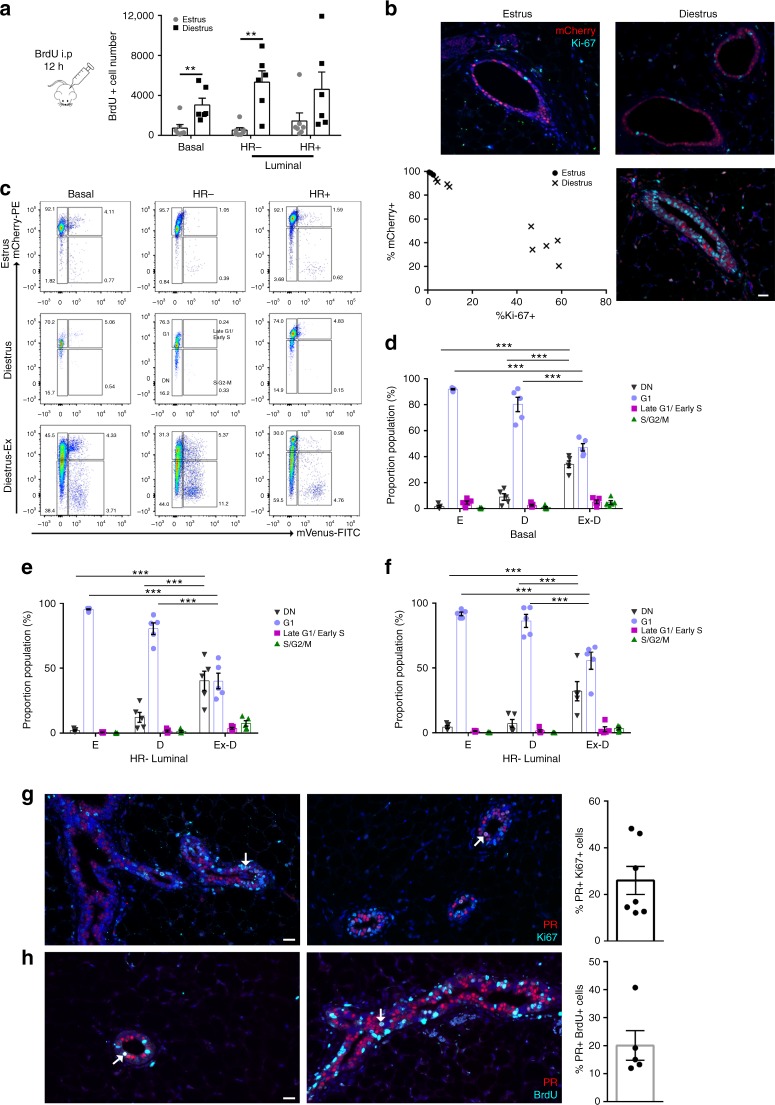
Diestrus heterogeneity in the mammary gland. **a** Flow cytometry analysis calculating the absolute number of BrdU+ cells from mammary epithelial subsets at estrus (*n* = 7) or diestrus (*n* = 6) showing a threefold (HR+) to ninefold (HR−) higher proliferation index at diestrus. **b** Representative immunofluorescent images of Ki-67+mCherry+ cells from an estrus, low-proliferative diestrus and a high-proliferative diestrus mammary gland. Scale bar = 25 µm, and quantification of Ki-67+ and mCherry+ cells in mammary glands from mice in estrus (*n* = 3) and diestrus (*n* = 9). **c** Representative FACS plot from Fucci2 mammary glands from estrus, non-proliferative diestrus and proliferative diestrus mice showing the proportion of cells in different phases of the cell cycle in basal, HR− and HR+ luminal populations. Bar graphs indicate the relative proportion of each cell cycle population within the (**d**) basal, (**e**) HR−, and **f** HR+ compartments from mice in estrus (**e**; *n* = 5), diestrus (**d**; *n* = 5) and high-proliferative diestrus (D-Ex; *n* = 5) stages (mean ± SEM). Two-way ANOVA analysis with Bonferroni correction used to determine significance between cell cycle phases in the basal, HR− and HR+ populations. *** represents statistical significance of *P* < 0.001 of D-Ex compared to estrus and diestrus stages. **g** Immunofluorescent images of PR+Ki-67+ cells from proliferating ductal and lobule structures. Arrows indicate PR+Ki-67+ cells. Scale bar = 25 µm and quantification of PR/Ki-67 expression (*n* = 7). **h** Immunofluorescent images of PR+ cells during S-phase in lobule and ductal structures of normal proliferating mammary glands. Arrows indicate PR+BrdU+ cells. Scale bar = 25 µm and quantification of PR/BrdU expression (*n* = 5)

These findings prompted us to further characterise the patterns of proliferation that occur within an estrous cycle. It is well known that progesterone is a mitogenic stimulant for the mammary gland and levels peak during diestrus phase of the estrous cycle^1,15^. However, diestrus can vary in duration^16^. BrdU (5-bromodeoxyuridine) was injected into mice during estrus or diestrus, 12 h before collecting the mammary glands and flow cytometry analysis was performed. As expected^2,15^, most cell division in mammary epithelial cells occurs during the diestrus phase; however, the number of BrdU+ cells observed during diestrus varied (Fig. 4a). Mammary glands collected from Fucci2 mice at estrus contained minimal Ki-67+ (Fig. 4b) or mVenus+ (S/G2/M) cells (Supplementary Fig. 4a). In contrast, mammary glands taken from mice in diestrus showed increased number of proliferating cells such that approximately half of the epithelial cells in both ductal and lobule structures were Ki-67+ (Fig. 4b, Supplementary Fig. 4a-b). Surprisingly, not all diestrus mice displayed this proliferating phenotype (Fig. 4b). Quantification of Ki-67+ and mCherry+ cells thus demonstrated that two states of diestrus exist; one with low proliferative events, similar to estrus and the other in an expansion state containing significant Ki-67+ cells (Fig. 4b). FACS analysis showed that sorted Fucci2 mammary cells were residing predominantly in G1 during estrus (Fig. 4c), and the non/low-proliferative diestrus state similarly consisted of cells in G1. On the other hand, the expansion state had majority of cells as DN or in S/G2/M (Fig. 4c–f). As a third to almost half of epithelial cells were residing in G1 we next wanted to determine whether these cells had proliferative capacity, or were differentiated. Basal (keratin 5 expressing cells) and HR− cells (Elf5+ cells) have been previously shown to have substantial progenitor and stem capacity^4,17–20^. It has been shown that the HR+ cells are maintained by their own lineage^21,22^, however it is not known if these HR+ cells expressing ER/PR could actively proliferative. Administering BrdU to mice for 12 h and performing tissue staining, we observed that BrdU positive mammary cells could express PR. However, the majority of PR+BrdU+ cells had variable to low expression of ER (Supplementary Fig. 4c), suggesting that ER expression may not be the best marker to determine HR+ proliferative capacity. Determining the percentage of HR+ proliferating cells, we stained the highly proliferating glands with PR and Ki-67 and observed that 26% ± 6% of all Ki-67+ cells expressed PR in both ducts and lobules (Fig. 4g). As Ki-67 expression is detected in several cell cycle phases, we took mammary glands that had been exposed to BrdU derivatives for a total of 4 h. Enabling us to query whether HR+ differentiated cells in S-phase were able to proliferate. We again observed that 20% ± 5.3% of BrdU+ cells expressed PR in ductal and lobule structures (Fig. 4h), indicating that HR+ differentiated luminal cells do have the capacity to enter into the proliferative cycle.

### Proliferative expansion does not occur every estrous cycle

**Fig. 5:**
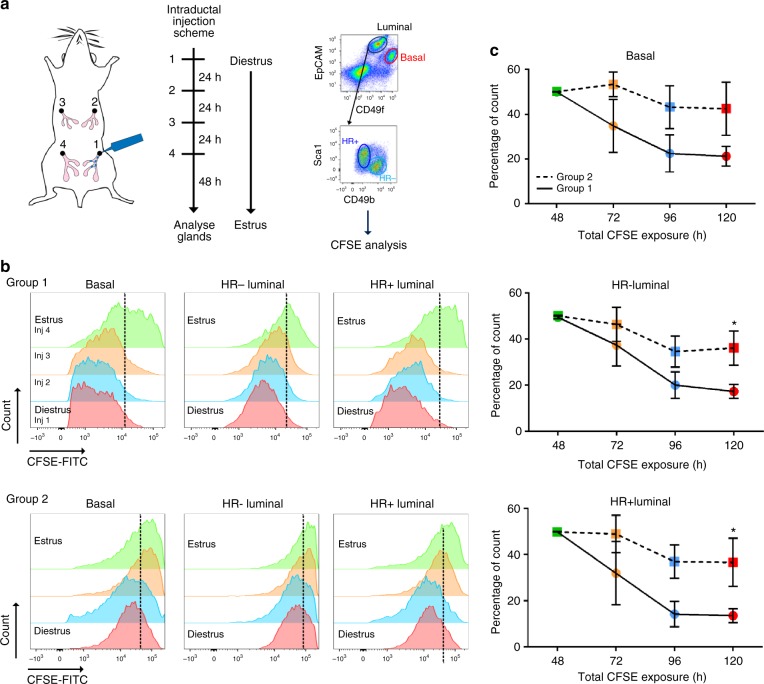
Two distinct diestrus stages occur in the mammary gland. **a** Schematic of workflow: Daily sequential injection of mammary glands with CFSE starting at the beginning of diestrus, to analyse in vivo cell division by CFSE dilution. **b** Histogram overlays show flow cytometry analysis of in vivo CFSE dilution, representing proliferation of basal, HR− and HR+ luminal cells within one estrous cycle. The green histograms show the CFSE intensity in mammary epithelial cells 48 h after injection 1, representing time-point zero (*t* = 0). The black dotted lines correspond to the median MFI of *t* = 0. The upper panels depict group 1, a high-proliferative estrous cycle, and the lower panels show group 2, a low proliferative estrous cycle. Histogram distributions were offset vertically for easy visualization. **c** Line diagrams represent the percentage of CFSE high events after subsequent injections calculated for each group (mean ± SEM, group 1 *n* = 3–5, group 2 *n* = 3). Student’s *t*-test analysis was performed. * represents statistical significance of *P* < 0.05

In order to deduce whether the expansion state occurs during every diestrus, we tracked the amount of proliferation the mammary gland undergoes during 1 estrous cycle. We used CFSE to trace dividing cells as CFSE fluorescence intensity is reduced following cell division, where CFSE was given via mammary intraductal injection at a non-toxic dose (Supplementary Fig. 4d-f). Mice were staged daily to identify the beginning of diestrus when the first injection was administered to a single 4th inguinal gland. This was followed by three injections into individual glands (Fig. 5a), and mammary glands analysed 48 h post last injection. Estrous stage was recorded throughout the entire assay (Tables 1, 2). The estrous cycle naturally fluctuates and we could observe two cycle lengths; group 1 had a longer diestrus (3/4 days) while group 2 had a shorter diestrus length (2 days). In vivo CFSE dilution time course showed group 1 containing substantial proliferation during a full estrous cycle as the intensity of CFSE is greatly reduced, whereas group 2 had fewer proliferative events due to CFSE intensities not changing significantly (Fig. 5b). Approximately 50% of the mammary epithelial cells in group 1 had reduced MFI compared to ~10% in group 2 (Fig. 5c). Thus, while expansion of the mammary gland may not occur during every estrous cycle, expansion is extremely strong driving up to 50% of all epithelial cells into division.

**Table 1:** Estrous stages of mice from group 1 at the time of each injection

Injection	Diestrus	Proestrus	Estrus	Metestrus	Next Diestrus
1	5				
2	5				
3	5				
4	1	3			
Analysis		1	4

**Table 2:** Estrous stages of mice from group 2 at the time of each injection

Injection	Diestrus	Proestrus	Estrus	Metestrus	Next Diestrus
1	3				
2	2	1			
3		1	2		
4			3		
Analysis				2	1

## Discussion

The decision to enter into the proliferative state in the mammary gland has thus far remained elusive and characterising the mammary cell cycle would reveal dynamics key to regeneration and differentiation. Specific cues are required for epithelial cells to enter into the cell cycle, and these may be cell type dependent. As the mammary gland relies on circulating hormone level fluctuations to trigger proliferation, the majority of proliferation occurs during the high-progesterone state, diestrus (Fig. 4a)^2,3^. The progesterone dominant luteal phase of the menstrual cycle in humans has shown variation not just in progesterone serum levels^23^, but also in the extent of proliferation observed^6,24^. Our tracking of cells over one reproductive cycle revealed that at any given time, only a subset of mice contained a highly proliferative phenotype and that substantial mammary epithelial expansion did not occur every estrous cycle. When expansion does occur, it is dramatic and encompasses ~50% mammary epithelial compartment. This study exposes the proliferative heterogeneity in the adult mammary gland, following the progesterone-dependent chain of molecular events.

At the single cell level, we demonstrate that sets of known mammary genes had characteristic expression profiles for mature HR+ cells and stem/progenitor cells. Stem/progenitor enriched basal and HR− luminal mammary cells had sustained and constant mRNA levels, likely enabling re-entry into the cell cycle. A small subset of HR+ cells with increased association to hormone receptor gene expression could be detected, while other HR+ cells expressed lower hormone receptor levels during diestrus^25^. Our observed expression fluctuations evident in the HR+ luminal compartment was consistent with the concept that these cells require a reduction of key hormone receptor/associated genes in order to facilitate cell cycle entry. Loss-of-Gata3 protein or mRNA using transgenic mouse models has reported increased proliferation in mammary HR+ luminal cells^9^. Alongside Gata3, we observed that ER, PR, FoxA1, and Cyclin D1 expression was reduced in S/G2/M reflective of a shift from differentiation to proliferative state. Recent studies have challenged the dogma of non/low-proliferative potential of HR+ cells, reporting progenitor activity and unipotent lineage maintenance in these cells^4,21,22^. Human breast ER+ cells have been previously shown to be mutually exclusive with Ki-67+ cells^26–28^, although whether these HR+ luminal cells have variable ER expression throughout the cell cycle remains to be seen. We observed more consistent expression with PR, demonstrating HR+ luminal lineage proliferative activity. Overall, our study illuminates the broader transcriptional change HR+ cells utilise in order to proliferate, as well as the proliferative heterogeneity of the entire mammary epithelial compartment.

Proliferative heterogeneity could be related to the time spent in the different cell cycle phases. G1 length has been implicated in human pluripotency and mouse neuronal stem/progenitor potential^11,29^. Further, lengthening of the total cell cycle and in particular G1, was observed to occur more in differentiated versus undifferentiated breast lobule structures of pre-menopausal human breast tissue^30^. We show that a short G1 phase is indicative of progenitor activity in all epithelial cell populations, whereas lengthy G1 associates with a more differentiated phenotype. Furthermore, although G1 lengths in the adult mammary gland are generally longer than those reported for embryonic tissues, the concept of G1 lengths per se determining stem/progenitor activity applies to mature breast tissue. We postulate that G1 lengths are fluid in the constantly changing hormone milieu of the mammary gland. It is highly probably that the increase/decrease of hormonal changes of key HR+ gene expressions, such as ER and Gata3, results in altering G1 lengths, enabling HR+ luminal cells to enter into a proliferative state. High proliferative occasions, including expansion in diestrus and pregnancy result in shorter G1, while remainder of the time, G1 lengths is extended.

In summary, our study addresses the complexity of proliferative heterogeneity during the female reproductive cycle. By dissecting the changes that occur within the reproductive cycle and length of the mammary cell cycle phases, we begin to understand the dynamic nature and hormone triggered proliferation in the breast. Our findings bring an exciting prospect that the mammary gland may not undertake as many proliferative cycles as previously anticipated, yet the robust nature of these proliferative events may significantly impact mutational accumulation and/or cell cycle deregulation. We also observe this proliferative heterogeneity occurring in both ductal and lobule structures, suggesting that cancerous cells in human breast tissues may arise from structures that are highly proliferative. Given the collective number of menstrual cycles has been identified as a breast cancer risk factor^5,31^, it remains to be seen whether the number of high-proliferation cycles could impact upon mutational accumulation and contribute towards cancer progression. As these studies were conducted in mice, and there are significant structural and hormonal differences between mouse and human breast tissues^32^, future work needs to address whether similar proliferative heterogeneity and differences in menstrual cycles are observed in human breast cells. Clarity is needed to determine whether increased breast cancer risk is attributed to the collective number of menstrual cycles or attributed to the total number of high proliferative cycles combined with the lifetime exposure of progesterone.

## Methods

### Mice

Experiments used virgin adult (10+ week old) female wild-type C57Bl/6 (OCI) and R26p-FUCCI2 (Riken Acc. No. CDB0203T)^7^ mice. All experiments were performed according to guidelines from the Canadian Council for Animal Care and under protocols approved by the Animal Care Committee of the Princess Margaret Cancer Centre, Toronto, Canada.

Estrous staging was determined by cytological characteristics of vaginal smears. A vaginal flush was performed with 50 μL of sterile PBS, then spotted onto a microscope slide, adhered for 10 min and visualised under a light microscope. Estrous staging was based on the presence and/or proportion of nucleated epithelial cells, cornified cells and lymphocytes^33,34^.

### Dissociation of mammary tissue into single cell suspension

The number 3 and/or number 4 mammary glands (lymph node removed) from female virgin mice were manually minced for 1 min and then enzymatically dissociated for 1.5 h in DMEM/F12 (1:1) supplemented with 2 mg mL^−1^ collagenase (Roche) and 200 U/mL hyaluronidase (Sigma). Samples were briefly vortexed every 30 min. The mammary glands were then processed to single cells as previously described^35^.

### Preparation of cells for flow cytometry

Single mammary cells were then incubated with the following primary antibodies: CD31-biotin (clone 390, eBioscience), CD45-biotin (clone 30-F11, eBioscience), Ter119-biotin (clone Ter119, eBioscience), EpCAM (clone G8.8, BioLegend), CD49f (clone GoH3, BioLegend), CD49b (HMα2, BioLegend), and Sca1 (clone D7, BioLegend). Biotin conjugated antibodies were detected with Streptavidin-eFluor450 (eBioscience). Cells were then filtered through a 30-μm cell strainer (Partec) and incubated with 4’, 6-diamidino-2-phenylindole (DAPI; Invitrogen) and were analysed by using an LSRII (Becton Dickinson), or sorted on a FACSAria II (Becton Dickinson). The gating strategy to select luminal and basal subsets is shown in Supplementary Fig. 5a. Flow cytometry data were analysed using FlowJo (version 10. Tree Star Inc.)

### Nucleoside incorporation studies

BrdU, CldU, and IdU (Sigma) were dissolved in PBS to a concentration of 10 mg mL^−1^.

BrdU incorporation: BrdU administered IP 12 h prior to tissue collection. After required duration of exposure, mice were culled and mammary glands digested as described above. For intracellular staining, cells were first stained with the indicated surface markers and then fixed with BD Cytofix/Cytoperm Buffer (BD Bioscience) for 20 min at 4 °C, followed by incubation with BD Cytoperm Plus Buffer for 10 min at 4 °C and re-fixed for 5 min at 4 °C. Cells were then treated with DNase (1 mg mL^−1^, Sigma) in PBS and then immunostained with anti-BrdU-FITC (clone 3D4, BioLegend).

CldU/IdU labelling: Adult female mice were given daily subcutaneous injections of 1 mg progesterone (Sigma) for 4 days, to mimic a high proliferative state. On day 4, mice received a single CldU IP injection followed by a single IdU IP injection either 3 or 14 h later. Mice were culled 1 h after IdU injection and mammary glands collected for histology analysis.

### CFSE

Carboxyfluorescein succinimidyl ester (CFSE, Molecular Probes, Thermo Fisher) fluorescent dye was dissolved in DMSO as 5 mM stock solution. For dye labelling, CFSE was resuspended to a final concentration of 150 µM in 0.2% Evans Blue/PBS. Female mice were staged daily 1 week prior to injections, and throughout the assay to ensure estrous cycling. On day of first injection only mice that were in metestrus the previous day and showed signs of early diestrus were included. CFSE was injected intraductally into the left 4, left 3, right 3, and right 4 mammary glands on sequential days. Individual glands were analysed by flow cytometry 2 days after the final injection.

### Mammary CFC assay

For CFC assays 500 sorted HR− luminal or basal cells and 1000 h+ mammary cells were seeded in complete FAD media (DMEM/F12 +180 μM adenine + 1.8 mM calcium), 10% FBS (Gibco), 0.5 μg mL^−^^1^ hydrocortisone (Sigma), 100pM cholera toxin (Enzo Life Sciences), 10 ng mL^−1^ epidermal growth factor (EGF, Peprotech), 5 μg mL^−1^ insulin (Gibco), 10 μM Y-7632 (Sigma), 50 μg mL^−1^ gentamicin (Sigma) in the presence of irradiated feeders and cultured for 7 days at 37 °C in a hypoxic (5% O_2_ and 5% CO_2_) incubator. For HR+ cells the TGFβ inhibitors SB431542 and RepSox (Sigma) were added to culture media at 10 and 25 µM, respectively, 2 days after plating. At the end of the assays, the colonies were fixed with acetone/methanol (1:1), stained with Giemsa (Fisher Scientific), and enumerated under a microscope.

To stimulate proliferation in CFC assays, 30 ng mL^−1^ R-Spondin I and Wnt3 (R&D Systems) was added to day 5 cultures. After 24 h colonies were trypsinised and cells analysed by flow cytometry.

### Culture for live imaging

Sorted basal, HR+, HR− luminal R26p-Fucci2 cells were grown on Eppendorf Cell Imaging 24-well plates with cover glass. After 4 days, cells were imaged in phenol red free complete FAD media in a 5% CO_2_/37 °C incubation chamber on an AxioObserver microscope (Zeiss) with a ×20/0.8 DIC Plan-Apochromat objective (Zeiss) at 20 min intervals for 24 h. Green (470 nm) and red (545 nm) signals were acquired in dual camera mode. The 2D images were processed by Zen 2.3 (Blue edition) software (Zeiss). All images were analysed by Image J (FIJI-64 bit) software. G1 was measured as the time between the first frame without fluorescence (after cell division) and the last frame with red fluorescence. S/G2/M phase was measured as the time between first and last frame with green fluorescence. This includes a short interval of S phase in which the cells were both red and green.

### Immunostaining and microscopy in tissue sections

Freshly isolated intact mammary glands were fixed in 4% PFA and processed into paraffin. A total of 4 µm sections were deparaffinised in xylene, gradually rehydrated in descending concentrations of ethanol, and subsequently treated in Borg Decloaker antigen retrieval solution (pH 6) for 30 min at 121 °C and 10 s at 90 °C using a Decloaking chamber (Biocare Medical). The samples were preblocked in PBS with 1% BSA and 0.1% Tween 20, before overnight incubation at 4 °C with primary antibodies: anti-BrdU (clone BU1/75, Abcam), anti-BrdU (Clone B44, BD Biosciences), anti-ERα (6F11, Novocastra), anti-PR (H190, Santa Cruz), anti-Keratin 5 (polyclonal, Abcam), anti-DsRed (polyclonal, Clontech), anti-GFP (polyclonal, Abcam), anti-Ki-67 (clone: SolA15, Thermo Fisher). The secondary antibodies were goat anti-mouse AF647, goat anti-rabbit Cy3 (Jackson Labs), goat anti-rat AF488 and/or goat anti-chicken AF488 (Invitrogen). Secondary antibody alone was used as a control. Sections were mounted with ProLong Gold antifade with DAPI (Invitrogen).

### Image analysis

Tissue sections were imaged using the Olympus BX50 microscope (Olympus) with tiling capacity and one-third to half of the mammary epithelium was imaged. After acquisition, images were stitched together in order to obtain a single image of the tissue. The individual channels were merged and displayed with Image J (FIJI-64 bit) software. All images were analysed by Image J software. One to two sections per sample were used for analysis. Cell were identified as being positive visually (using a secondary only control to determine positivity) and the Cell Counter tool in Image J used.

Ts and Tc quantification: Between 255 and 920 CldU+ and/or IdU+ cells from seven mice and between 374 and 877 CldU+ and/or IdU+ cells from five independent biological samples were analysed, respectively.

PR-Ki-67 quantification: Between 430 and 1085 cells from seven mice were analysed.

PR-BrdU quantification: 150–920 BrdU+ cells were analysed from five mice.

Estrus and Diestrus quantification: 805–2914 cells were analysed from 3 to 9 independent biological samples, respectively. (Diestrus phase is between 2 and 3 days^36,37^. To robustly cover the diestrus phase, three independent biological replicates from the early, mid, and late diestrus phase were collected.)

For all quantification of images, reference cells were DAPI+ nuclei epithelia cells.

### RNA extraction and single cell RT-PCR

Single-cell gene-expression was determined using Fluidigm’s 96.96 qPCR Dynamic Array microfluidic chips. A total of 96 cells containing G1, G1 high, S/G2/M and DN cells from the basal, HR− luminal and HR+ luminal populations from two pooled biological replicates were sorted directly into individual wells of 96-well PCR plates containing 9 µL of lysis and preamplification mix: 5 µL of CellsDirect PCR mix (Invitrogen), 0.2 µl of SuperScript-III RT/Platinum Taq mix (Invitrogen), 1.0 µL of a mixture of all pooled primer assays (500 nM each), and 2.8 µL of DNA suspension buffer (TEKnova). After sorting, plates were placed into a thermocycler for combined reverse transcription (50 °C for 15 min, 95 °C for 2 min) and target-specific amplification (20 cycles; each cycle: 95 °C for 15 s, 60 °C for 4 min). cDNA was diluted 1:5 with TE before qPCR on the BioMark HD. cDNA synthesis and amplification were performed according to Fluidigm real-time PCR Dynamic Array IFC Protocol (BioMark Fluidigm). All primers were validated via prior to inclusion. Hierarchical clustering, PCA, violin plots, ANOVA, and gene expression analysis were performed using the SINGuLAR Analysis Toolset 2.1 (Fluidigm). Genes were clustered together using the Pearson method, and samples are clustered together using the Euclidean method.

### Ts and Tc calculations

The length of S phase (Ts) and total cell cycle length (Tc) were calculated according to Brandt et al.^38^. Briefly, to determine Ts or Tc, CldU+ and IdU+ cells were counted. Ts was calculated by the ratio of cells that left S phase during the 3 h exposure using the following formula:

$${\mathrm{Ts}} = {\mathrm{CldU}} + {\mathrm{IdU}} - {\mathrm{ cells}}/{\mathrm{CldU}} + {\mathrm{cells}} = {\mathrm{3}}\,{\mathrm{hours}}/{\mathrm{Ts}}.$$

To determine Tc, an interval between Ts and Tc – Ts, but no longer than 2 × Tc – Ts was selected and the length of the cell cycle was calculated using the following formula:

$${\mathrm{Tc}} = {\mathrm{14}}\,{\mathrm{hours}} + {\mathrm{(Ts}}\,{\mathrm{x}}\,\left( {{\mathrm{IdU}}^ + {\mathrm{CldU}}^ - {\mathrm{cells}}/{\mathrm{IdU}}^ + {\mathrm{cells}}} \right).$$

G2/M-phase can be calculated by assuming it is shorter than 15 h, (time after IdU exposure for Tc) minus Ts (7.4 h). This the approximate duration of G2/M is between 4 h and 7.6 h (~5.8 h). G1 is calculated by subtracting the lengths of Ts (7.4 h) and G/2/M (~5.8 h) from Tc (20.1 h). Thus G1 is ~6.9 h.

### Statistics and reproducibility

Data are presented as the mean of at least three independent experiments with S.E.M, unless a different number of repeats is stated in the legend where *P*-values and *N* numbers are indicated in the figure legends. Comparisons between multiple groups were analysed using analysis of variance (ANOVA) followed by a multiple comparisons test, or a Student’s *t*-test as indicated in the figure legends GraphPad Prism v. 6.0. Statistical significance is indicated as follows: *p < 0.05, **p < 0.01 and ***p < 0.001. No statistical method was used to predetermine sample size. No animals were excluded from the study. No method of randomization was used. The investigators were not blinded to allocation during experiments or outcome assessment.

### Data availability

The datasets generated during and/or analysed during the current study are available from the corresponding author on request.

## Electronic supplementary material

Supplementary Information

## References

[1] Fata JE, Chaudhary V, Khokha R (2001). Cellular turnover in the mammary gland is correlated with systemic levels of progesterone and not 17beta-estradiol during the estrous cycle. Biol. Reprod..

[2] Joshi PA (2010). Progesterone induces adult mammary stem cell expansion. Nature.

[3] Asselin-Labat ML (2010). Control of mammary stem cell function by steroid hormone signalling. Nature.

[4] Giraddi RR (2015). Stem and progenitor cell division kinetics during postnatal mouse mammary gland development. Nat. Commun..

[5] Pike MC, Spicer DV, Dahmoush L, Press MF (1993). Estrogens, progestogens, normal breast cell proliferation, and breast cancer risk. Epidemiol. Rev..

[6] Navarrete MAH (2005). Assessment of the proliferative, apoptotic and cellular renovation indices of the human mammary epithelium during the follicular and luteal phases of the menstrual cycle. Breast Cancer Res..

[7] Abe T (2013). Visualization of cell cycle in mouse embryos with Fucci2 reporter directed by Rosa26 promoter. Development.

[8] Asselin-Labat ML (2007). Gata-3 is an essential regulator of mammary-gland morphogenesis and luminal-cell differentiation. Nat. Cell Biol..

[9] Kouros-Mehr H, Slorach EM, Sternlicht MD, Werb Z (2006). GATA-3 maintains the differentiation of the luminal cell fate in the mammary gland. Cell.

[10] Lange C, Calegari F (2010). Cdks and cyclins link G_1_ length and differentiation of embryonic, neural and hematopoietic stem cells. Cell Cycle.

[11] Pauklin S, Vallier L (2013). The cell cycle state of stem cells determines cell fate propensity. Cell.

[12] Coronado D (2013). A short G1 phase is an intrinsic determinant of naïve embryonic stem cell pluripotency. Stem Cell Res..

[13] Joshi PA (2015). RANK signaling amplifies WNT-responsive mammary progenitors through R-SPONDIN1. Stem Cell Rep..

[14] Zeng YA, Nusse R (2010). Wnt proteins are self-renewal factors for mammary stem cells and promote their long-term expansion in culture. Cell Stem Cell.

[15] Beleut M (2010). Two distinct mechanisms underlie progesterone-induced proliferation in the mammary gland. Proc. Natl Acad. Sci. USA.

[16] Gardner RL (1991). Rugh’s Mus redux. The Mouse; Its Reproduction and Development.

[17] Shehata M (2012). Phenotypic and functional characterisation of the luminal cell hierarchy of the mammary gland. Breast Cancer Res..

[18] Prater MD (2014). Mammary stem cells have myoepithelial cell properties. Nat. Cell Biol..

[19] Rios AC, Fu NY, Lindeman GJ, Visvader JE (2014). In situ identification of bipotent stem cells in the mammary gland. Nature.

[20] Van Keymeulen A (2011). Distinct stem cells contribute to mammary gland development and maintenance. Nature.

[21] Van Keymeulen A (2017). Lineage-restricted mammary stem cells sustain the development, homeostasis, and regeneration of the estrogen receptor positive lineage. Cell Rep..

[22] Wang C, Christin JR, Oktay MH, Guo W (2017). Lineage-biased stem cells maintain estrogen-receptor-positive and -negative mouse mammary luminal lineages. Cell Rep..

[23] Wang J (2013). Comment on “progesterone/RANKL is a major regulatory axis in the human breast”. Sci. Transl. Med..

[24] Masters JRW, Drife JO, Scarisbrick JJ (1977). Cyclic variation of DNA synthesis in human breast epithelium. J. Natl Cancer Inst..

[25] Pal B (2017). Construction of developmental lineage relationships in the mouse mammary gland by single-cell RNA profiling. Nat. Commun..

[26] Clarke RB, Howell A, Anderson E (1997). Estrogen sensitivity of normal human breast tissue in vivo and implanted into athymic nude mice: analysis of the relationship between estrogen-induced proliferation and progesterone receptor expression. Breast Cancer Res. Treat..

[27] Shoker BS (1999). Estrogen receptor-positive proliferating cells in the normal and precancerous breast. Am. J. Pathol..

[28] Hopkinson BM (2017). Establishment of a normal-derived estrogen receptor-positive cell line comparable to the prevailing human breast cancer subtype. Oncotarget.

[29] Li VC, Ballabeni A, Kirschner MW (2012). Gap 1 phase length and mouse embryonic stem cell self-renewal. Proc. Natl Acad. Sci. USA.

[30] Russo, J. & Russo, I. H. in *Cellular and Molecular Biology of Mammary Cancer* 399–417 (Springer, US, 1987).10.1007/978-1-4613-0943-7_22

[31] Chavez-MacGregor M (2005). Postmenopausal breast cancer risk and cumulative number of menstrual cycles. Cancer Epidemiol. Biomark. Prev..

[32] Dontu G, Ince TA (2015). Of mice and women: a comparative tissue biology perspective of breast stem cells and differentiation. J. Mammary Gland Biol. Neoplasia.

[33] Nelson JF, Felicio LS, Randall PK, Sims C, Finch CE (1982). A longitudinal study of estrous cyclicity in aging C57BL/6J mice: I. Cycle frequency, length and vaginal cytology. Biol. Reprod..

[34] Caligioni, C. NIH Public Access. 1–11 (2010). 10.1002/0471142301.nsa04is48.Assessing

[35] Prater, M., Shehata, M., Watson, C. J. & Stingl, J. Enzymatic Dissociation, Flow Cytometric Analysis, and Culture of Normal Mouse Mammary Tissue. (eds. Helgason, C. & Miller, C.). In *Basic Cell Culture Protocols. Methods in Molecular Biology (Methods and Protocols)*. Vol. 946 (Humana Press, Totowa, NJ) (2013). 10.1007/978-1-62703-128-8_25. 10.1007/978-1-62703-128-8_25 23179846

[36] Cora MC, Kooistra L, Travlos G (2015). Vaginal cytology of the laboratory rat and mouse. Toxicol. Pathol..

[37] Byers SL, Wiles MV, Dunn SL, Taft RA (2012). Mouse estrous cycle identification tool and images. PLoS ONE.

[38] Brandt MD, Hübner M, Storch A (2012). Brief report: adult hippocampal precursor cells shorten S-phase and total cell cycle length during neuronal differentiation. Stem Cells.

