# Supplementary material for: Proliferative heterogeneity of murine epithelial cells in the adult mammary gland
Source: Commun Biol. 2018 Aug 13;1:111. doi: 10.1038/s42003-018-0114-7 (PMC6123670; doi:10.1038/s42003-018-0114-7)
Supplement: Supplementary file 1 — Supplementary Information [file 42003_2018_114_MOESM1_ESM.pdf]

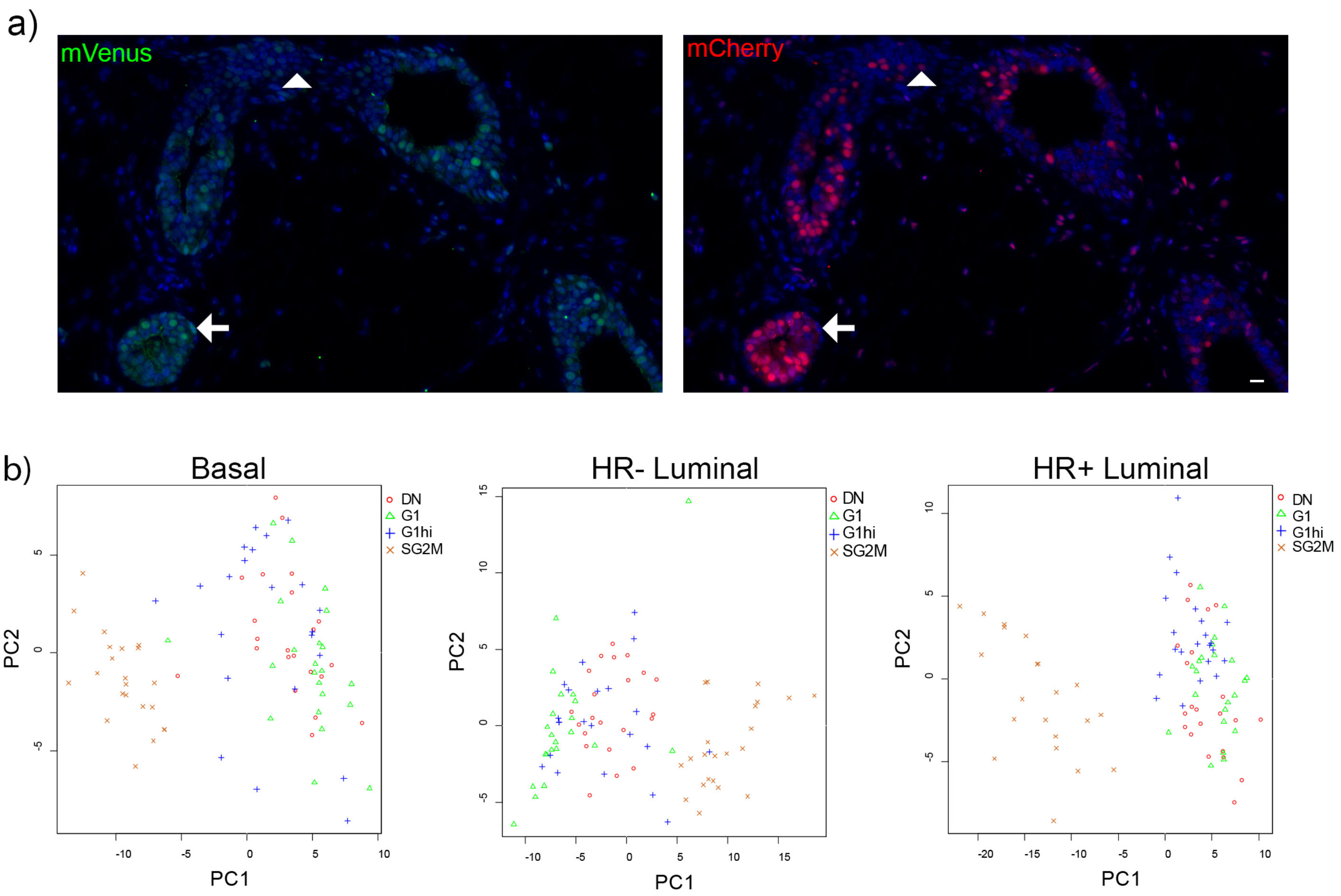

Supplementary Figure 1. Mammary glands from Fucci2 mice.
a) Immunofluorescence staining for mCherry to detect cells in G1 (arrowhead) and mVenus to detect S/G2/M (arrows) phases of the cell cycle in basal cells. Scale bar = 25 µm. b) PCA plots show DN, G1, G1hi and S/G2/M cells in the mammary epithelial subtypes.

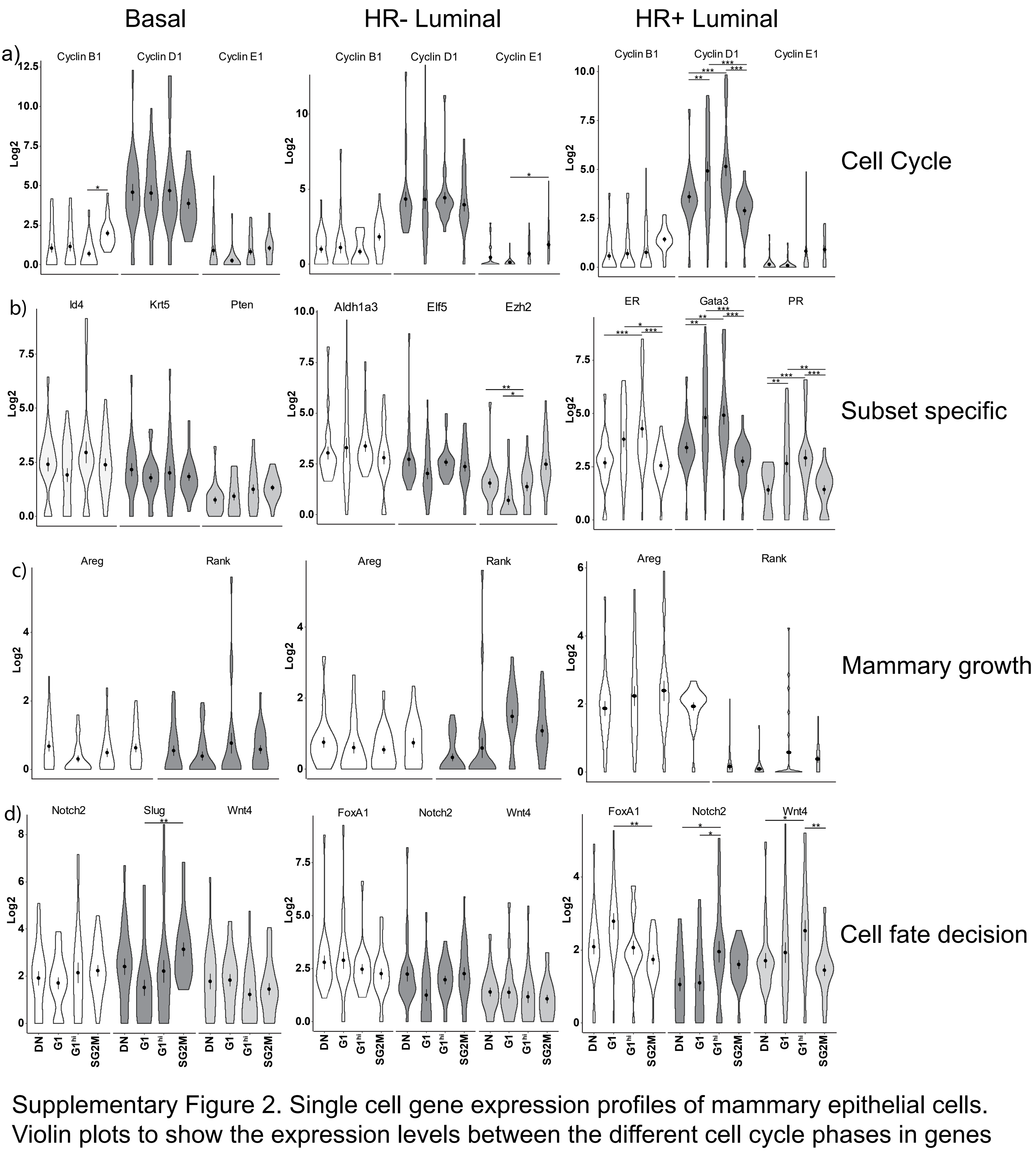

Supplementary Figure 2. Single cell gene expression profiles of mammary epithelial cells. Violin plots to show the expression levels between the different cell cycle phases in genes relating to a) Cell cycle, b) Subset specific, c) Mammary growth and d) Cell fate decision genes. 2-way ANOVA analysis with Tukey's correction used to determine significance. *, **, *** represents statistical significant at $P < 0.05$, $P < 0.01$, $P < 0.001$ respectively.

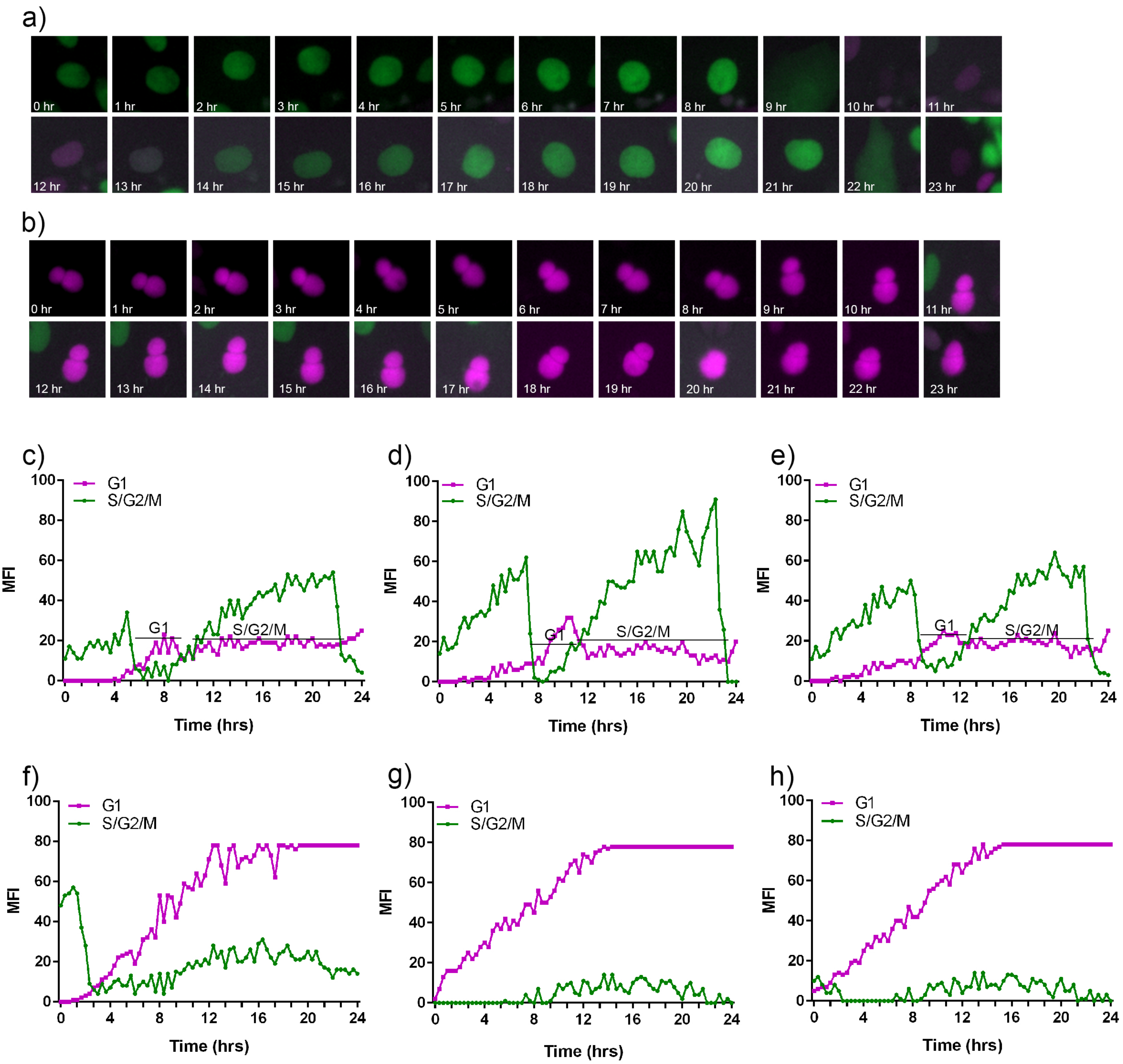

Supplementary Figure 3. Monitoring the cell cycle and cell cycle length. Representative time-lapse images of the cell cycle determined by tracing Fucci2 fluorescence from a) a cycling cell, where black lines indicate the intersect of magenta: green to indicate G1 or S/G2/M length and b) a cell with extended G1. Changes in G1 (magenta) or S/G2/M (green) fluorescence intensity during 24 hour imaging in a representative proliferative c) basal d) HR- luminal or e) HR+ luminal cell and a representative differentiated f) basal, g) HR- luminal or h) HR+ luminal cell.

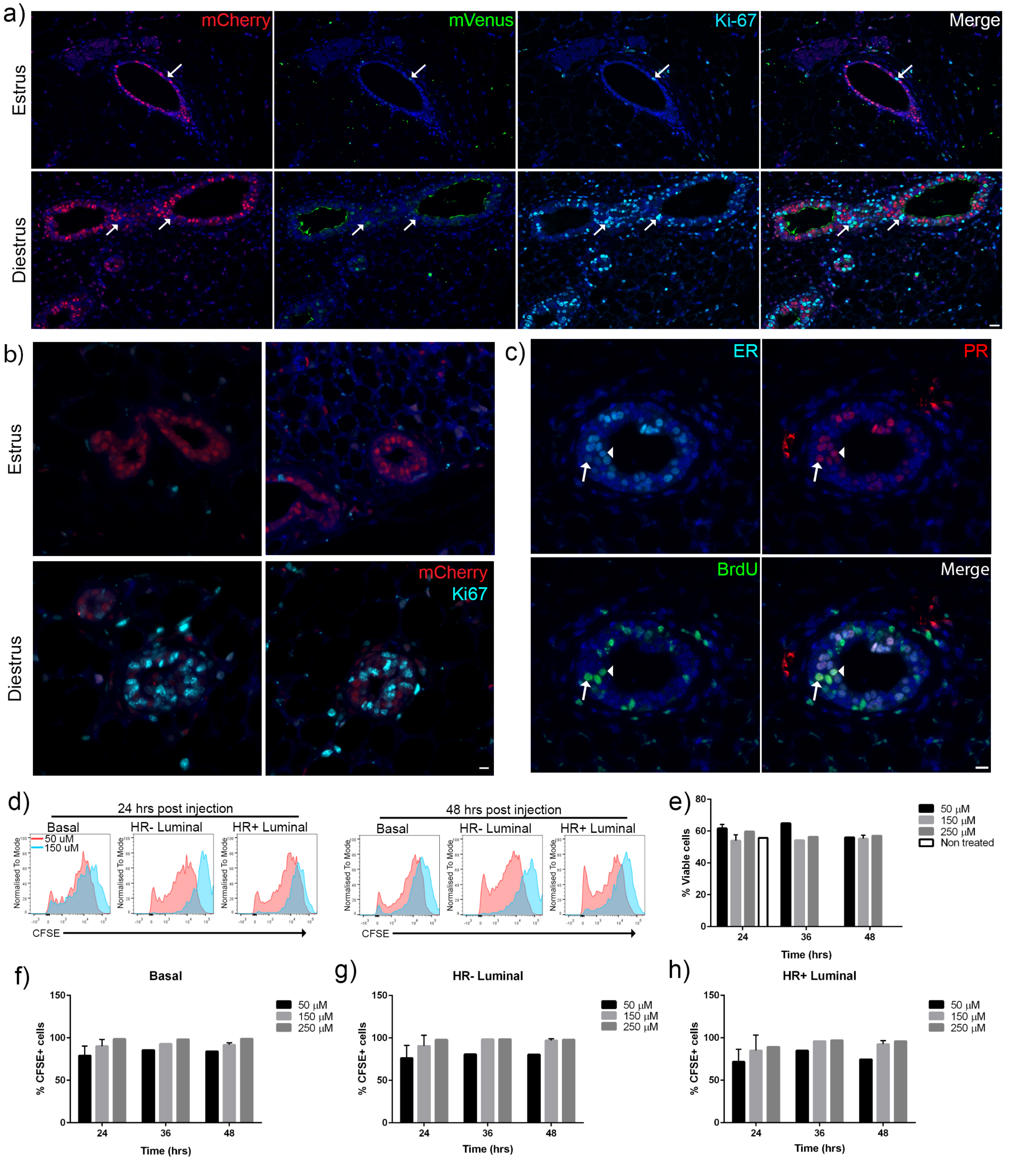

Supplementary Figure 4. Minimal toxicity of in vivo CFSE administration.
a) Immunofluorescent images of mCherry (red), mVenus (green) and Ki-67 (cyan) positive cell in ductal structures of Fucci2 mammary gland from an estrus mouse (upper panels) or a diestrus mouse (lower panels). Arrows indicate mVenus+ Ki-67+ cells. Scale bar = 25 µm. b) Immunofluorescent images of mCherry (red) and Ki67 (cyan) cells in lobule structures of Fucci2 mammary glands from estrus (upper panels) or diestrus (lower panels) mice. c) Immunofluorescent image of ER, PR and BrdU expression. Arrows indicate PR+ -/low ER expression in a proliferating (BrdU+) cell. Arrowhead indicates PR+ER+ proliferating cell. Scale bar = 25 µm. d) Histograms showing the MFI for two different doses of CFSE uptake at 24 and 48 hours post injection. e) Bar graph of the percentage live cells after various doses and time exposures showing minimal toxicity of CFSE to mammary epithelial cells. Bar graphs showing the percentage CFSE positive cells by flow cytometry at 24, 36 and 48 hours after injection of 20 µl of the indicated concentration of CFSE, for f) basal, g) HR- luminal and h) HR+ luminal cells.

a)

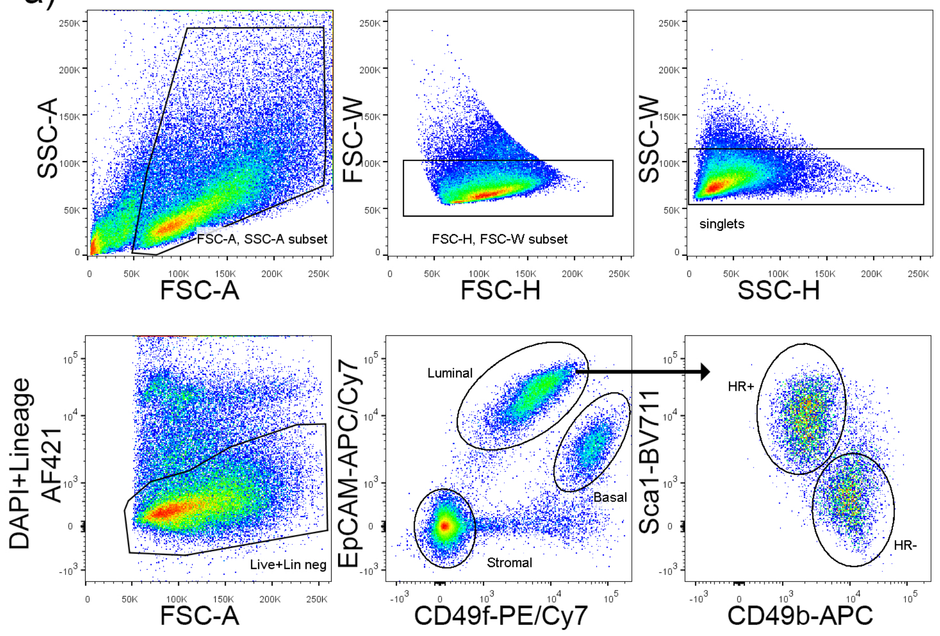

Supplementary Figure 5. Gating strategy.
a) Flow cytometry gating strategy of dissociated mammary epithelial cells showing basal (EpCAMloCD49fhi) and luminal (EpCAM+CD49flo) populations followed by further segregation into hormone positive (HR+, Sca1+CD49b-) and hormone negative (HR-, Sca1-CD49b+) luminal subpopulations.
